# Thyroid dysfunction (TD) induced by PD-1/PD-L1 inhibitors in advanced lung cancer

**DOI:** 10.1016/j.heliyon.2024.e27077

**Published:** 2024-02-24

**Authors:** Yanling Wang, Xiaoxuan Yang, Jia Ma, Shenglan Chen, Ping Gong, Ping Dai

**Affiliations:** a School of Medicine, Shihezi University, Shihezi, Xinjiang, 832000, People's Republic of China; b Department of Oncology, The Third Affiliated Hospital of School of Medicine of Shihezi University, Shihezi, Xinjiang, 832000, People's Republic of China; c Department of Radiotherapy, Shanghai Fourth People's Hospital, School of Medicine, Tongji University, Shanghai, 200434, People's Republic of China; d Department of General Surgery, Shanghai Jian Gong Hospital, Shanghai, 200434, People's Republic of China; e Department of Molecular Radiation Oncology, German Cancer Research Center (DKFZ), Neuenheimer Feld 280, 69120, Heidelberg, Germany

**Keywords:** Thyroid dysfunction, Efficacy, Immune checkpoint inhibitors, PD-1/PD-L1 inhibitors, Advanced lung cancer

## Abstract

**Background:**

Thyroid Dysfunction (TD) is a common immune-related adverse events (irAEs) in the treatment of advanced lung cancer with programmed cell death protein 1 (PD-1) and programmed death 1 ligand (PD-L1) inhibitors, with incidence accounting for 6–8% of all irAEs. The incidence of TD is receiving increasing attention from clinicians, given its potential impact on clinical efficacy. However, the molecular mechanisms, biomarkers, and clinical impact of TD resulting from PD-1/PD-L1 inhibitor treatment in advanced lung cancer are unclear.

**Objective:**

To present a comprehensive review of current advancements in research about the molecular mechanisms, influential factors, and clinical manifestations in the treatment of advanced lung cancer with PD-1 and PD-L1 inhibitors, as well as the correlation between TD and the efficacy of PD-1 and PD-L1 inhibitors.

**Methods:**

A systematic search was conducted using PubMed, Web of Science, Cochrane Library, Embase and Google Scholar databases, with the keywords including thyroid dysfunction, efficacy, mechanisms, immune checkpoint inhibitors, PD-1/PD-L1 inhibitors, and advanced lung cancer.

**Results:**

PD-1/PD-L1 inhibitors can induce T cell-mediated destructive thyroiditis, thyroid autoantibody-mediated autoimmunity, and a decrease in the number of immunosuppressive monocytes (circulating cluster of differentiation (CD)14^+^ human leukocyte antigen (HLA)-DRlow/negatives monocytes, CD14^+^ HLA-DR ^+ lo/neg^), leading to TD. Several factors, including peripheral blood inflammatory markers, body mass index (BMI), baseline thyroid-stimulating hormone (TSH) level, gender, smoking history, hypertension, and previous opioid use, may also contribute to the development of TD. However, there is currently a lack of reliable predictive biomarkers for TD, although anti-thyroid antibodies, TSH levels, and peripheral blood inflammatory markers are expected to be predictive.

Interestingly, some studies suggested a positive correlation between TD and clinical efficacy, i.e., patients experiencing TD showed better outcomes in objective response rate (ORR), disease control rate (DCR), progression-free survival (PFS), and overall survival (OS), compared with those without TD. However, most of these studies were single-center and had small sample sizes, so more multi-center studies are needed to provide further data support.

**Conclusion:**

TD resulting from PD-1/PD-L1 inhibitor treatment in advanced lung cancer may be associated with good clinical outcomes. The clarification of the molecular mechanisms underlying TD and the identification of reliable predictive biomarkers will guide clinicians in managing TD in this patient population.

## Abbreviations

TD: Thyroid dysfunction
PD-1: Programmed cell death protein 1
PD-L1: Programmed death 1 ligand
CD: Circulating cluster of differentiation
HLA: Human leukocyte antigen
CD14^+^HLA-DR ^+ lo/neg^ monocytes: CD14^+^ HLA-DRlow/negative monocytes
BMI: Body mass index
TSH: Thyroid-stimulating hormone
ORR: Objective response rate
DCR: Disease control rate
PFS: Progression-free survival
OS: Overall survival
ICI: Immune checkpoint inhibitor
IrAE: Immune-related adverse event
IARC: International Agency for Research on Cancer
HR: Hazard rate
CI: Confidence interval
Th: Helper T cells
IL: Interleukin
NK cell: Natural killer cell
MHC-II: Major histocompatibility complex II
IFN-γ: Interferon gamma
DC: Dendritic cells
Tregs: Regulatory T cells
G-CSF: Granulocyte colony-stimulating factor
GM-CSF: Granulocyte-macrophage colony-stimulating factor
TCR: T-cell receptor
FGF2: Fibroblast growth factor 2
TNF-α: Tumor necrosis factor-α
FT4: Free thyroxine
FT3: Free triiodothyronine
rT3: Anti-T3
NLR: Neutrophil/lymphocyte ratio
PLR: Platelet/lymphocyte ratio
PNI: Prognostic nutrition index
ICP: Immune checkpoint
CSCO: Chinese Society of Clinical Oncology
NSCLC: Non-small cell lung cancer
ED-SCLC: Extensive-stage small cell lung cancer
EP: Etoposide + Cisplatin
EC: Etoposide + Carboplatin
AP: Pemetrexed + Platinum
RR: Risk ratio

## 1 Introduction

Immune checkpoint inhibitors (ICIs) have been increasingly used as immunotherapy for cancer, but immune-related adverse events (irAEs) often occur. Of particular concern is immune-related thyroid dysfunction (TD), accounting for approximately 6–8% of the total irAEs [1]. TD refers to disorders with abnormal thyroxine secretion caused by programmed cell death protein (PD-1) and programmed death 1 ligand (PD-L1) inhibitors treatment. Some studies have found that the occurrence of TD may be associated with good outcomes [[2], [3], [4]], but no consistent conclusions have been reached. Therefore, exploring the molecular mechanisms and the possible predictors of its occurrence is of great significance, which can help to timely identify TD to avoid the development of secondary adverse events such as thyroid storm and myxedema coma. In addition, further study of the correlation between TD and efficacy will help select populations suitable for immunotherapy, and provide a reference for the development of individualised treatment plans for patients.

According to the latest data released by the International Agency for Research on Cancer (IARC), the incidence of lung cancer is on a declining trend globally, but it still ranks second, accounting for 11.4% of all new cancer cases. Besides, it has the highest mortality rate [5]. Due to the lack of specific tests and symptoms in the early stages of lung cancer, more than 60% of patients are diagnosed at an intermediate to advanced stage or have metastasis, and the 5-year survival rate is less than 5% [6]. Traditional treatment options include chemotherapy and targeted therapy. However, the 5-year survival rate of chemotherapy is only 20–30%, with serious adverse effects, such as bone marrow suppression and impairment of liver and kidney function [7]. Although targeted therapy can increase the overall survival (OS) (mOS not reached *vs* 17 months) compared with chemotherapy [8], it is susceptible to drug resistance, which generally occurs 9–14 months after treatment [9,10].

The use of ICIs in the treatment of advanced lung cancer is revolutionary. Among ICIs, PD-1/PD-L1 inhibitors have been widely used in the treatment of advanced lung cancer, and showed good efficacy due to their long-lasting effect, tolerable toxicity and wide range of applications. Compared with chemotherapy, the use of ICIs in the treatment of advanced lung cancer significantly prolonged patients' OS (Hazard rate (HR) = 0.60, 95% confidence interval (CI) [0.41–0.89], *p* = 0.005) and progression-free survival (PFS) (mPFS: 10.3 months *vs* 6.0 months, HR = 0.50, 95% CI [0.37–0.68], *p* < 0.001) and greatly improved patients' quality of life [11]. However, several studies have shown that treatment with ICIs can lead to irAEs in multiple systems, such as the respiratory, endocrine, and digestive systems [12]. Although most irAEs are self-limiting, sometimes serious irAEs still occur, affecting the course and efficacy of the treatment and even the survival of patients [13].

In clinical practice, the majority of TD resulting from the use of PD-1/PD-L1 inhibitors in the treatment of advanced lung cancer is mild (grade I - II) [14], and most patients have no significant clinical symptoms. Previous study [15] showed that the patients who developed TD (TD (+))had better outcome in mOS and mPFS (29.8 months *vs* 8.1 months (*p* < 0.001) and 8.7 months *vs* 1.8 months (*p* < 0.01)) and prognosis compared to the patients who without TD (TD (−)) This may be due to that the occurrence of TD may strongly activate the immune system. Meanwhile, the study also showed no difference in ORR and DCR between different degrees of TD (*p* > 0.05) [15].

## 2 Methodology

A systematic search of databases (PubMed, Web of Science, Cochrane Library, Embase and Google Scholar) was conducted to collect relevant articles with published up to December 2023. The collected articles were analyzed to extract data on molecular mechanisms, possible influencing factors, and predictive biomarkers leading to TD, as well as correlation between TD and clinical efficacy of PD-1/PD-L1 inhibitor treatment. Thyroid dysfunction, efficacy, mechanisms, ICIs, PD-1/PD-L1 inhibitors, and advanced lung cancer were used as the keywords. The Science Slides plug-in for Microsoft PowerPoint was used for drawing.

## 3 Molecular mechanisms and clinical manifestations of TD caused by PD-1/PD-L1 inhibitors therapy

### 3.1 Molecular mechanisms, influencing factors and possible predictive markers for the occurrence of TD

**Fig. 1:**
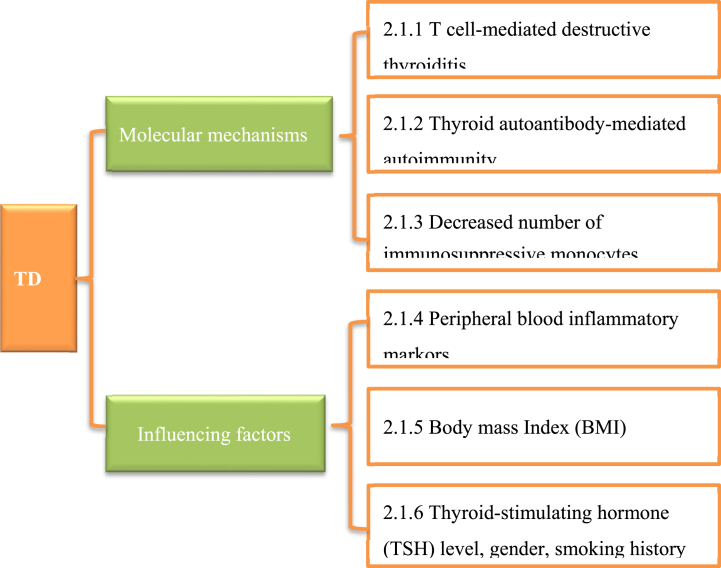
Molecular mechanisms and influencing factors of thyroid dysfunction (TD).

The use of both PD-1 and PD-L1 inhibitors in the treatment of malignancies may lead to TD. The mechanism of TD's occurrence involves factors such as T cell-mediated destructive thyroiditis, thyroid autoantibody-mediated autoimmunity, and reduced number of immunosuppressive monocytes (CD14^+^ HLA-DR ^+ lo/neg^ monocytes). In addition, it may also be associated with peripheral blood inflammatory markers, BMI, and TSH levels. However, there is a lack of valid and convenient clinical biomarkers to predict the development of TD when receiving immunotherapy (Fig. 1).

#### 3.1.1 T cell-mediated destructive thyroiditis

It has been shown that the pattern of TD following the PD-1 inhibitor treatment is consistent with destructive thyroiditis [16]. Kotwal et al. [17] reported that a predominance of CD8^+^, PD1^+^ and CD4^−^CD8^−^T lymphocytes in the thyroid was found in patients with anti-PD-1/PD-L1-induced TD. Double-negative (CD4^−^CD8^−^) lymphocytes contain large numbers of γ-δT lymphocytes, a type of self-reactive pro-inflammatory effector cell that has been shown to infiltrate inflamed tissue and cause organ damage [18]. Besides, increased expression of PD-1 on T cells in thyroid tissue in patients with TD may be a cause of thyroid susceptibility. Some researchers have found that helper T cells and regulatory T cells (Tregs) may play a role in the development of thyroiditis [19]. When thyrotoxicosis occurs during immunotherapy, T cells can proliferate and differentiate towards helper T cells, which further activate B cells, convert B cells into plasma cells and increase the secretion of autoantibodies, thus causing an autoimmune response [20].

##### 3.1.1.1 Correlation between cytokine levels and TD

In addition, changes in the levels of various cytokines are highly associated with TD occurrence [21,22]. Interleukin-2 (IL-2), mainly produced by helper T cells 1 (Th1), has multiple effects on various immune systems [23]. It promotes the differentiation of initial CD4^+^ T cells into Th1 cells and helper T cells 2 (Th2), as well as the cytotoxicity of CD8^+^ T cells and natural killer cells (NK cells) [24]. Some studies [22,25] have found that higher levels of IL-2 not only induce the binding of major histocompatibility complex II (MHC-II) to thyroid autoantigens, but also enhance the ability of CD8^+^ T cells to kill the thyroid gland, inducing thyroid autoimmunity. Interferon gamma (IFN-γ) is one of the key factors involved in various immune responses. It is produced by Th1 cells, NK cells, NKT cells, etc. Its secretion is promoted by IL-2. IFN-γ participates in the activation of dendritic cells (DC) and monocyte macrophages [26], enhancing cellular immunity. IFN-γ can also activate the signaling of PD-1 axis and suppress cellular immunity by up-regulating the expression of PD-L1. However, high-level PD-L1 expression in tumour patients is beneficial for anti-PD-1/PD-L1 therapy [27]. Scholars [28] found that the number of CD4^+^ Th1 cells secreting IL-2 and IFN-γ increased after PD-1 inhibitor treatment, and the levels of pro-inflammatory factors IL-2 and IFN-γ also increased. Therefore, this may be one of the reasons for the occurrence of TD after anti-PD-1 treatment. IL-10 is a cytokine with anti-inflammatory and immunomodulatory functions and produced by Tregs [29], and PD-1 is involved in regulating the proliferation and differentiation of Tregs [21]. It has been found that IL-10 levels decreased after anti-PD-1 treatment, perhaps due to the loss of energy of Tregs [30]. The decrease in IL-10 levels may also be related to the emergence of thyroid autoantibodies [21,31]. In summary, anti-PD-1 treatment could induce thyroid autoimmunity by increasing the levels of pro-inflammatory factors (such as IL-2, IFN-γ) released by helper T cells, or by suppressing the level of IL-10 in Tregs. In addition, it has been found that the level of granulocyte colony-stimulating factor (G-CSF) decreases and that of granulocyte-macrophage colony-stimulating factor (GM-CSF) increases in the patients who developed TD after the use of ICIs [21]. Therefore, the changes in the levels of G-CSF and GM-CSF may also contribute to the occurrence of TD. Besides, previous study has shown that G-CSF is positively correlated with Th2 cytokine levels, suggesting an increase in the Th1/Th2 ratio during the development of TD.

#### 3.1.2 Thyroid autoantibody-mediated autoimmunity

Osorio et al. [32] found that almost all cases of TD caused by anti-PD-1 therapy were accompanied by the presence of thyroid autoantibodies. Therefore, anti-thyroid antibodies may be a hematological biomarker for TD. Anti-PD-1 therapy is usually considered to be T cell-mediated immunity, but antibody production suggested the involvement of B cells and humoral immunity [17,19,32]. This implies that anti-PD-1 therapy not only affects T cell-mediated immunity but also regulate humoral immunity, which inconsistent with previous results [16,17]. Relevant studies have shown that PD-1 is highly expressed in activated B cells and regulated B cells through both T cell-independent [33] and T cell-dependent mechanisms [34], which is associated with antibody production. Hollowell et al. [35] found that thyroid autoantibodies were detectable in up to 11% of healthy subjects, but only about 4% of patients were diagnosed with autoimmune thyroiditis, suggesting that the autoantibody levels in some individuals were below the onset level, remaining quiescent in the absence of PD-1 blockade or other immune interference [36].

Although thyroid autoimmunity is suspected to be associated with disruption of self-tolerance [37], the mechanism by which anti-PD-1 therapy regulates this autoimmunity remains unclear. Mazarico et al. [38] observed that patients who developed TD after the use of ICIs resulting in TD had significantly lower levels of thyroid autoantibodies compared with patients with autoimmune thyroiditis. This suggests that there may be difference in pathogenesis between ICIs-induced thyroiditis and autoimmune thyroiditis [38]. Similar conclusions were reached for anti-PD-L1 therapy in a study by Kotwal et al. [39]. Therefore, the clarification of the impact of anti-PD-1 and anti-PD-L1 on humoral immunity is important for understanding the aetiology of TD and optimizing treatment strategies.

#### 3.1.3 Decreased number of immunosuppressive monocytes (CD14^+^ HLA-DR ^+ lo/neg^ monocytes) increases the risk of autoimmunity

Danae et al. [40] reported that the immune cell phenotype of patients with PD-1 inhibitor-induced thyroiditis was special compared with that of patients with autoimmune thyroiditis. The PD-1 levels did not differ significantly between patients with autoimmune thyroiditis and healthy volunteers, but there was a significant increase in NK cell and T cell subsets, including CD4^+^ T helper cells, CD8^+^ cells Toxic T cells, γ-δT cells and NKT cells. Compared with patients with autoimmune diseases, patients with PD-1 inhibitor-induced thyroiditis showed special differences in immune cell phenotype, and showed a decrease in the number of immature NK cells (CD56^+^CD16^−^) as well as CD14^+^ HLA-DR ^+ lo/neg^ monocytes. Human leukocyte antigen DR (HLA-DR) is an MHC-class II molecule widely expressed on B lymphocytes, monocytes, macrophages, activated T lymphocytes, activated NK lymphocytes, and human progenitor cells. Its function is to present antigens uptake by antigen presenting cells to T-cell receptors (TCRs), which leads to the activation of T-cells [41] and the completion of a variety of immune responses. However, tumours can escape recognition by the immune system through the down-regulation of MHC-II molecules [42]. Gustafson et al. [43] found that bone marrow cells in tumour patients were affected by the tumour and its microenvironment, leading to increased expression of suppressive monocytes such as CD14^+^ monocytes. Tumours could down-regulate the expression of HLA-DR on CD14^+^ monocytes by some mechanism, turn CD14^+^ monocytes into highly suppressive monocytes and promote their survival by secreting various cytokines such as GM-CSF, fibroblast growth factor 2 (FGF2), and IL-1β [43,44]. Monocytes with reduced or no expression of HLA-DR are called CD14^+^ HLA-DR ^+ lo/neg^ monocytes, and its high expression is closely associated with the development of a variety of tumours such as pancreatic cancer [44], renal carcinoma [43], invasive paediatric sarcoma [45], and ovarian cancer [46]. Studies [43,44,47] have shown that increased expression of CD14^+^ HLA-DR ^+ lo/neg^ monocytes in cancer patients inhibits T cell proliferation and DC maturation through a variety of mechanisms, leading to immune escape. The reduction of such monocytes and the increased expression of HLA-DR molecules when PD-1/PD-L1 inhibitors are applied to treat cancer lead to reactivation of the immune system, which may increase the risk of autoimmunity. It has been reported that thyroid autoantigens are homologous to tumour-associated antigens, and both present on the surface of antigen-presenting cells together with HLA, participating in T and B cell immunity. This may be potentiated by PD-1 inhibitors by increasing the expression of HLA, thus inducing thyroid autoimmunity [21].

**Fig. 2:**
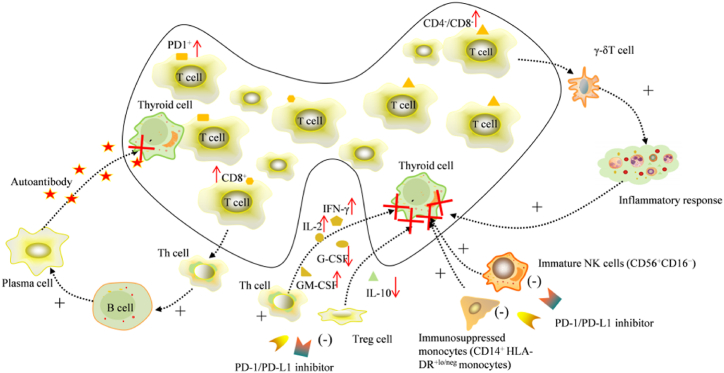
Molecular mechanisms for the occurrence of TD induced by PD-1/PD-L1 inhibitors in the treatment of advanced lung cancer (adapted from Refs. [[17], [18], [19], [20], [21],^28^,^30^,^40^]).

In summary, after using PD-1/PD-L1 inhibitors to treat advanced lung cancer CD8^+^, PD1^+^ and CD4^−^CD8^−^T lymphocytes increase in the thyroid gland of patients who develop TD compared with those of patients without TD. In addition, the production of thyroid autoantibodies suggests that humoral immunity is also involved in the development of TD, and a decrease in NK cells (CD56^+^CD16^−^) and CD14^+^ HLA-DR ^+ lo/neg^ monocytes contributes to this process. The Th cells and Tregs are also involved in this process by increasing IL-2, IFN-γ and GM-CSF or decreasing cytokines (IL-10 and G-CSF). The molecular mechanisms of TD induced by PD-1/PD-L1 inhibitors in the treatment of advanced lung cancer are shown in**（**Fig. 2**）**.

#### 3.1.4 Correlation of TD with peripheral blood inflammatory markers

Siddiqui et al. [48] found that the development of thyroiditis was closely related to inflammatory activity. TSH was significantly associated with the inflammatory markers IL-6 and Tumor Necrosis Factor-α (TNF-α). However, free thyroxine (fT4) was only associated with TNF-α, and free triiodothyronine (fT3) was negatively associated with IL-6. Another study [49] found that C-reactive protein was significantly associated with anti-T3 (rT3), an inactive thyroid hormone negatively correlated with T3. In addition, Yamauchi et al. [16] pointed out that immune-related TD was essentially a form of thyroiditis, the development of which was closely related to the involvement of inflammatory factors, so immune-related TD might have similar mechanisms to the development of thyroiditis.

Inflammatory markers in peripheral haematology, such as neutrophil/lymphocyte ratio (NLR), platelet/lymphocyte ratio (PLR) and prognostic nutrition index (PNI) are often used to reflect the body's active inflammation and inflammatory depletion. Besides, PNI is also used to assess nutritional status and immune status. Several studies [19,50,51] have shown that NLR, PLR and PNI can be used as independent predictors of irAEs. Saeka Egami et al. [52] showed that NLR >2.3 and PLR >4.5 could indicate a significantly increased risk of developing almost all irAEs when PD-1 inhibitors were used to treat non-small cell lung cancer. Liu et al. [53] showed that low NLR and PLR were significantly associated with irAEs in the treatment of lung cancer using PD-1. In addition, Peng et al. [54] reported that the PNI significantly higher in patients who developed irAEs than in patients without irAEs.

In general, immune-related TD is one of the most common endocrine-related toxicities induced by PD-1/PD-L1 inhibitors in lung cancer. And the NLR, PLR, and PNI are expected to be predictive biomarkers for immune-related TD.

#### 3.1.5 Correlation of TD with body mass index (BMI)

Rena et al. [55] found that the risk of TD continued to increase with the increase of BMI when ICIs were applied to treat lung cancer. For every 1 kg/m^2^ increase in weight, the risk of TD increased by 10.0%. However, Han-sang et al. [56] found that there was no difference in TD between patients with different BMI. The overall BMI was high in the study of Rena et al. conducted in Israel (approximately one in five being obese), and low in the study of Han-sang et al. conducted in South Korea (only 2.6% samples had a BMI >30). Therefore, it is speculated that their different conclusions may be caused by ethnic factor. Cao [57] found that in obese patients, increased levels of adipokines (e.g., leptin) and cytokines (e.g., TNF-α and IL-6) lead to Th1/Th2 imbalance and promote inflammation and autoimmune diseases. These pro-inflammatory factors may contribute to the development and progression of TD. Marzullo et al. [58] found an increased prevalence of TD and thyroid autoantibodies in obese individuals, which might be associated with increased leptin levels. The study by Rena et al. [55] also found that higher BMI was correlated more with thyrotoxicosis and TD occurred significantly earlier in the overweight and obese groups than in the normal weight group. This indicates a stronger immune response to PD-1/PD-L1 inhibitors in patients with higher BMI.

#### 3.1.6 Other factors related to the TD

A single-center retrospective clinical trial by Rena et al. [59] found that baseline TSH >2.19 μL was significantly associated with the development of TD during anti-PD-1 treatment of lung cancer. Another study also showed that high baseline TSH levels were associated with the development of hypothyroidism in the context of immunotherapy for melanoma [60], but the mechanism was unknown. In addition, some studies have also suggested that TD was more common in female patients [30,61]. This may be related to the different effects of estrogen and androgen on the immune response [30], although the mechanism has yet to be clarified. Furthermore, Zhan et al. [31] found that the incidence of TD associated with ICIs (manifesting as hypothyroidism) was related to the type of malignancy, with a higher incidence in lung cancer and melanoma. But, further research is needed to clarify if there is difference for different types of lung cancer. Kim et al. [62] has explored that smoking history, concomitant hypertension and previous opioid use were associated with irAEs-associated TD, showing a positive association between smoking history, hypertension and TD and a negative association between opioid use and TD. This may be due to the fact that smoking and hypertension upregulate PD-L1 expression, enhancing immune function [63,64], whereas opioid use suppresses immune function [65]. The mechanism is also needed to be further investigated. In addition, direct cell killing of NK cells, inflammatory intermediate monocytes during immunotherapy [17], and complement-mediated inflammatory responses may also be associated with TD [12].

### 3.2 Clinical manifestations of TD

IrAEs-related TD is a common side effect of PD-1/PD-L1 inhibitor therapy. According to the results of several retrospective studies, the incidence of TD ranged from 7% to 21% after PD-1 inhibitor therapy and 6%–11% after PD-L1 inhibitor therapy. Real-world studies [2,39,66,67] showed that anti-PD-1/PD-L1 therapy led to a high incidence of TD (40% or even 50%), with TD occurring around 2–4 cycles of PD-1/PD-L1 inhibitor injections. There are various clinical manifestations of TD, among which the manifestations of hypothyroidism mainly includes constipation, cold tolerance and weight gain, and those of thyrotoxicosis mainly includes insomnia, palpitation, hyperhidrosis and heat tolerance. However, subclinical hypo-or hyperthyroidism generally has no specific symptoms [68]. There appear to be several types of TD caused by ICIs treatment, but in fact, these are different stages of the disease manifestation. It has been found that there is a uniform pattern of TD during anti-PD-1 and PD-L1 therapy, that is, most cases have an early onset, with a rapidly developing asymptomatic phase of thyrotoxicosis, followed by a rapid transformation to hypothyroidism that may require lifelong levothyroxine hormone replacement may be required [69]. This is consistent with the mechanism of destructive thyroiditis. Yoon et al. [70] reported that the presence of thyroid autoantibodies predicted progression to overt hypothyroidism. Early detection of TD is particularly important since the majority of TD induced by PD-1/PD-L1 inhibitor therapy has no obvious symptoms, and patients rarely seek active medical attention for TD. Most thyroiditis lasts about 6 months before it goes away on its own. About one-fifth develop permanent hypothyroidism that require replacement therapy, while patients with thyrotoxicosis need beta-blockers to prevent severe cardiovascular disease when they develop palpitations and tremors, and generally do not need to discontinue PD-1/PD-L1 inhibitors [71].

## 4 Mechanism of PD-1 and PD-L1 inhibitors and their application in advanced lung cancer

### 4.1 Mechanism of PD-1 and PD-L1 inhibitors

**Fig. 3:**
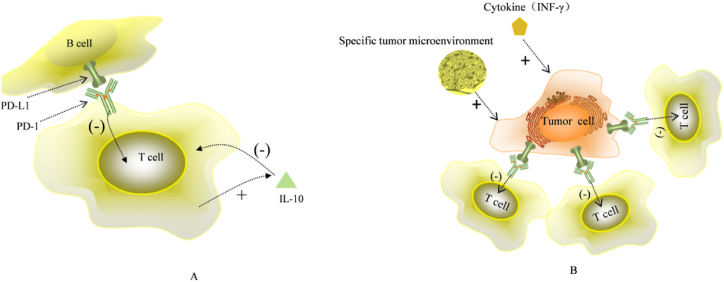
**(A)** Under normal circumstances, the combination of PD-1 and PD-L1 inhibits further activation of T cells, induces apoptosis of activated T cells, and produces large amounts of IL-10, further promoting immunosuppression as a means of maintaining self-tolerance and preventing autoimmunity (adapted from Ref. [81]). **(B)** PD-L1 expression on the surface of tumor cells is significantly upregulated by the combination of specific tumor microenvironment, various cytokines (IFN-γ) and chemokines, which can be used to achieve immune escape and thus reduce the anti-tumor immune response (adapted from Ref. [82]).

**Fig. 4:**
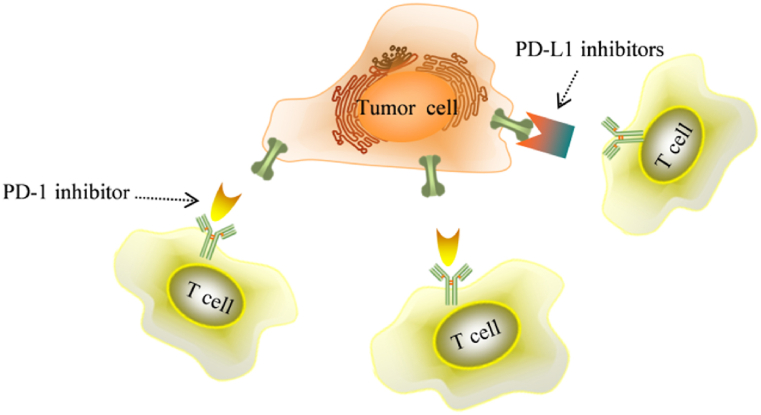
PD-1 and PD-L1 inhibitors work by binding PD-1 and PD-L1 respectively, blocking the binding pathway between PD-1 and PD-L1 and reactivating the T cells for anti-tumor effects (adapted from Ref. [83]).

Immune checkpoints (ICPs) are a group of transmembrane proteins that are expressed on the surface of immune cells and regulate the immune system. Their main role is to maintain self-tolerance and prevent autoimmunity. However, ICPs interfere with the protective immune response, allowing tumor cells to evade the immune system [72], leading to tumor growth and spread. The widely studied ICPs are PD-1 and its main ligand PD-L1, which are inhibitory co-stimulatory molecules that ensure the stability and integrity of immune function by negatively regulating the activation of T cells [73]. Anti-PD-1/PD-L1 drugs are monoclonal antibodies that inhibit the negative regulation of the immune response and suppress tumor growth and spread by reactivating T cells to kill tumor cells directly or indirectly [39]. As a key regulator of immune tolerance and immune depletion, PD-1 is widely expressed on the cell surface of the adaptive (i.e., activated T cells and B cells) and innate immune systems (i.e., NK cells and macrophages) [74]. It is worth noting that PD-1 is highly expressed on the surface of tumor-specific T cells (CD8^+^ T cells predominantly) [75,76]. PD-L1 is expressed on the surface of activated T and B cells, macrophages, endothelial cells, etc., especially in the context of inflammatory responses [77]. As a tumor-promoting factor [78], it is significantly upregulated on the surface of tumor cells in response to the specific tumor microenvironment and various cytokines (e.g., IFN-γ), and has been shown to be associated with multiple tumor progression [79,80]. Under normal conditions, PD-1 binding to PD-L1 inhibits further activation of T cells, induces apoptosis of activated T cells, and produces large amounts of IL-10, further promoting immunosuppression [81]. This protects tissues from damage caused by immune responses (Fig. 3A). Similarly, high expression of PD-L1 in tumor cells can help achieve immune escape, thus reducing the anti-tumor immune response [82] (Fig. 3B). Anti-PD-1 and PD-L1 drugs promote the activation of adaptive immunity and release anti-tumor immune responses by binding PD-1, and PD-L1 respectively, blocking the binding pathway between PD-1 and PD-L1 and reducing the inhibitory signal on T-cell activation [83] (Fig. 4).

### 4.2 PD-1 and PD-L1 inhibitor therapy in advanced lung cancer

PD-1/PD-L1 inhibitors have become the main treatment option for patients with advanced lung cancer, with the advantages of less systemic toxic side effects, lower drug resistance and better efficacy than chemotherapy. It can significant improve patient survival and quality of life [84]. Currently, many PD-1 and PD-L1 inhibitors have been recommended by Chinese Society of Clinical Oncology (CSCO) guidelines for the treatment of lung cancer at various stages **(**Table 1**)**. Studies [85,86] have shown that the use of PD-1 and PD-L1 inhibitors in the treatment of all types of advanced lung cancer or in combination with chemotherapy yields positive results, with ORR (20% *vs* 9%, *p* = 0.008), PFS (mPFS: 3.5 months *vs* 2.8 months), and OS (mOS: 9.2 months *vs* 6.0 months) significantly higher than those of chemotherapy [87,88].

**Table 1:** Examples of PD-1/PD-L1 inhibitors recommended by Chinese Society of Clinical Oncology (CSCO) guidelines for applications in advanced lung cancer.

Drugs	Type	Launch date (year)	Applications
Durvalumab/+ EP/EC	PD-L1	2017	For patients with stage III NSCLC after concurrent radiotherapy and first-line treatment of extensive-stage SCLC
Pembrolizumab ± AP	PD-1	2014	For first-line treatment of stage IV NSCLC
Carrelizumab + AP	PD-1	2018	For first-line treatment of Stage IV NSCLC
Sindilizumab + AP	PD-1	2018	For first-line treatment of Stage IV NSCLC
Nivolumab	PD-1	2014	For second-line treatment of Stage IV NSCLC
Atezolizumab/+ EC	PD-L1	2016	For first-line treatment of patients with Stage IV driver-free NSCLC and extensive-stage SCLC
**… …**			

NSCLC: Non-small cell lung cancer; ED-SCLC: Extensive-stage small cell lung cancer; EP: etoposide + cisplatin; EC: etoposide + carboplatin; AP: pemetrexed + platinum.

## 5 IrAEs

### 5.1 Correlation between TD and prior irAEs

According to a previous meta-analysis, the incidence of irAEs was 72% after PD-1 inhibitor treatment and 60% after PD-L1 inhibitor treatment [89]. IrAEs occur in all systems of the body such as common skin disorders (itching, rashes, etc.), endocrine disorders (hypothyroidism, hyperthyroidism, etc.), liver disorders (elevated alanine aminotransferase, albumin aminotransferase, hepatitis, etc.), gastrointestinal disorders (nausea, vomiting, colitis, etc.), respiratory disorders (coughing, dyspnea, pneumonia) and musculoskeletal disorders (arthralgia and myalgia) [90]. The incidence of grade III or higher adverse events after PD-1/PD-L1 inhibitor treatment is low, but lethal adverse events still occur [91]. Immune-related pneumonia is the most common fatal irAE, accounting for 35% of anti-PD-1/PD-L1-related deaths, followed by hepatitis (22%) and neurotoxic effects (15%) [92], whereas myocarditis is the most fatal irAE, with deaths occurring in approximately 50% of patients with myocarditis [93]. However, there are no studies on the relationship between priorly occurred other types of irAEs and the occurrence of TD.

### 5.2 Correlation of irAEs with outcome and prognosis

Interestingly, in several retrospective studies [61,94], PFS and OS were found to be significantly longer in the group of patients who experienced irAEs when treated with ICIs for a variety of advanced tumor. However, several studies [[95], [96], [97]] have found that the correlation between irAEs and good outcomes and prognosis after PD-1/PD-L1 inhibitor treatment depends on different tumor types. Specifically, positive correlations were found in renal-cell carcinoma, head and neck cancer and uroepithelial cancer, but no correlations were found in lung cancer, melanoma and gastrointestinal carcinoma [96]. Nevertheless, in a study of patients receiving nivolumab for progressive gastric cancer, patients with irAEs were found to have remarkably longer median PFS (7.5 months *vs* 1.4 months, *p* < 0.001) and OS (16.8 months *vs* 3.2 months, *p* < 0. 001) compared with group without irAEs [98]. Furthermore, in a meta-analysis of the correlation between irAEs and outcome in lung cancer, patients with irAEs were found to have a pooled relative risk ratio (RR) of 2.43 (95% CI [2.06–2.88], *p* < 0.00001) for the ORR compared with patients without irAEs, indicating that patients with irAEs had better outcomes. Besides, patients with irAEs had longer OS (HR = 0.51, 95% CI [0.43–0.61], *p* < 0.00001) and PFS (HR = 0.5, 95% CI [0.44–0.57], *p* < 0.00001) compared with patients without irAEs group [1,99,100].

## 6 Correlation between efficacy, prognosis, and TD induced by PD-1/PD-L1 inhibitor therapy of lung cancer

### 6.1 Correlation between outcome, prognosis, and TD

Wang et al. [101] found that not all irAEs were associated with good efficacy and prognosis in the treatment of advanced lung cancer with PD-1/PD-L1 inhibitors. Skin, endocrine, and gastrointestinal toxicity might be predictors of enhanced efficacy, but lung and hepatobiliary damage were not correlated with efficacy. TD is one of the most common endocrine-related toxicities associated with the use of PD-1/PD-L1 inhibitors in the treatment of advanced lung cancer, accounting for 6%–8% of irAEs [1]. However, recent studies have come to conflicting conclusions as to whether there is a correlation between outcome, prognosis and TD. Kim et al. [102] reported that among 58 patients with advanced lung cancer treated with PD-1 inhibitors, 19 patients developed TD and showed significantly higher DCR (15.8% *vs* 0.0%, *p* = 0.011) and ORR (31.6% *vs* 10.3%, *p* = 0.044) and longer PFS (mPFS: 118 days *vs* 61 days, *p* = 0.014) and OS (mOS not achieved, *p* = 0.025) compared with those without TD. Baek et al. [56] found that among patients with advanced lung cancer treated with PD-1/PD-L1 inhibitors, the TD group had longer treatment duration for ICIs (8.1 ± 8.0 months *vs* 4.0 ± 6.8 months, *p* < 0.001), median PFS (27.0 ± 2.3 months *vs* 18.0 ± 1.6 months) and OS (*p* = 0.003), compared with the no-TD group. Besides, the patients who developed TD had better activation of the immune system, more pronounced anti-tumor effects, and lower possibility of developing drug resistance and progression. However, Percik et al. [2] reported that there was a correlation between the occurrence of TD and good outcome and prognosis when PD-1/PD-L1 inhibitors were used to treat renal cancer (HR = 0.19, 95% CI [0.06–0.60], *p* = 0.005), whereas no correlation was observed in lung cancer. D'Aiello [4] and Wu et al. [103] also found no significant differences in treatment response and prognostic Indicators of survival between TD and no-TD groups when ICIs were used to treat lung cancer.

### 6.2 Differences in the efficacy and prognosis of PD-1/PD-L1 inhibitor therapy between different types of TD

Baek et al. [56] performed a subgroup analysis based on the type of TD and found a significantly improved prognosis in the new-onset overt hypothyroidism group compared with patients with previous thyroid disease and thyrotoxicosis (*p* = 0.002), and this was not related to the type of ICIs. Interestingly, patients with thyrotoxicosis had a higher RR for mortality compared with the no-TD group. Thyrotoxicosis is transient and subsequently progresses to hypothyroidism [69,70]. Baek et al. [56] reported that the prognosis of hypothyroidism was significantly improved after the use of ICIs compared with that of other types of TD. This suggests that the stimulatory effect of TSH might be associated with improved prognosis, but the mechanism needs to be further explored. Yu et al. [14] also found the differential relationships between TD subtypes and survival, specifically, the advanced lung cancer patients with dominant hyperthyroidism had the best OS followed by the patients with subclinical hyperthyroidism, dominant hypothyroidism and subclinical hypothyroidism. These findings differ from those of Baek et al. [56] study. However, Thuillier et al. [15] reported that there were no significant differences in treatment response and prognosis between different types of TD.

## 7 Conclusion and prospects

According to previous researches, the occurrence of thyroid dysfunction (TD) in patients with advanced lung cancer treated with PD-1/PD-L1 inhibitors has a strong correlation with their prognosis. Furthermore, various degrees of TD have different effects on prognosis. The prognosis of patients with advanced lung cancer in the new-onset overt hypothyroidism group is significantly better than that of patients with previous thyrotoxicosis. The mechanisms of TD induced by PD-1/PD-L1 inhibitors involve destructive thyroiditis mediated by T cells, autoimmunity mediated by thyroid autoantibodies, and reduced expression of immunosuppressive monocytes (CD14^+^ HLA-DR ^+ lo/neg^ cells). Interestingly, the occurrence of TD induced by the treatment with PD-1/PD-L1 inhibitors in advanced lung cancer may also be related to the patient's peripheral blood inflammatory markers, BMI, and TSH level, but the molecular mechanisms still need to be explored.

Overall, advances in PD-1/PD-L1 inhibitor treatment for advanced lung cancer have highlighted the need for managing immune-related TD. Additionally, in-depth studies on the relationships between PD-1/PD-L1 inhibitors and TD can improve care for lung cancer patients.

For exploring the correlation between TD and efficacy resulting from PD-1/PD-L1 inhibitors treatment for advanced lung cancer and the influencing factors, there are the following major challenges and opportunities:

(1) Most of the current studies on the correlation between TD and efficacy are retrospective, single-center clinical studies with small sample sizes. Therefore, they are prone to information bias, have poor representativeness, and are unable to rigorously demonstrate the causal relationship between TD and efficacy. More prospective, multicenter, and cohort studies are needed to valid the conclusions, which requires clinicians to closely monitor thyroid function and to collaborate in a multicenter setting.
(2) Although some studies suggest that baseline TSH levels, BMI, thyroid autoantibodies, and other factors are associated with the development of TD, the specific molecular mechanisms are still unclear. Besides, there is a lack of reliable predictive models to demonstrate whether there is a correlation. Therefore, further studies are necessary to explore reliable predictive biomarkers and molecular mechanisms, and to establish predictive models, which will help clinicians to detect TD in the early stage and to formulate personalized treatment plans.
(3) Although there have been certain results on the molecular mechanism of TD, most of them are speculations of researchers. Therefore, further clinical and basic studies are still needed.
(4) It is necessary to conduct further studies to explore the correlation between different types of TD and the efficacy.
(5) Whether there is a correlation between the priorly occurred other types of immune-related adverse events and the development of TD has not yet been reported, so further studies are needed.

## Availability of data statement

The datasets used and analyzed during the current study are available from the corresponding author on reasonable request.

## Funding statement

This study was supported by the 10.13039/501100001809National Natural Science Foundation of China (no. 81560381).

## CRediT authorship contribution statement

**Yanling Wang:** Writing – original draft, Software, Resources, Formal analysis, Data curation, Conceptualization. **Xiaoxuan Yang:** Resources. **Jia Ma:** Software, Resources. **Shenglan Chen:** Resources. **Ping Gong:** Supervision, Project administration, Investigation, Funding acquisition, Conceptualization. **Ping Dai:** Writing – review & editing, Supervision, Methodology, Investigation, Funding acquisition.

## Declaration of competing interest

The authors declare the following financial interests/personal relationships which may be considered as potential competing interests:Ping Gong reports financial support was provided by 10.13039/501100001809National Natural Science Foundation of China. If there are other authors, they declare that they have no known competing financial interests or personal relationships that could have appeared to influence the work reported in this paper.
